# Supplementary material for: Transcriptomic Insights into Mechanisms of Early Seed Maturation in the Garden Pea (Pisum sativum L.)
Source: Cells. 2020 Mar 23;9(3):779. doi: 10.3390/cells9030779 (PMC7140803; doi:10.3390/cells9030779)

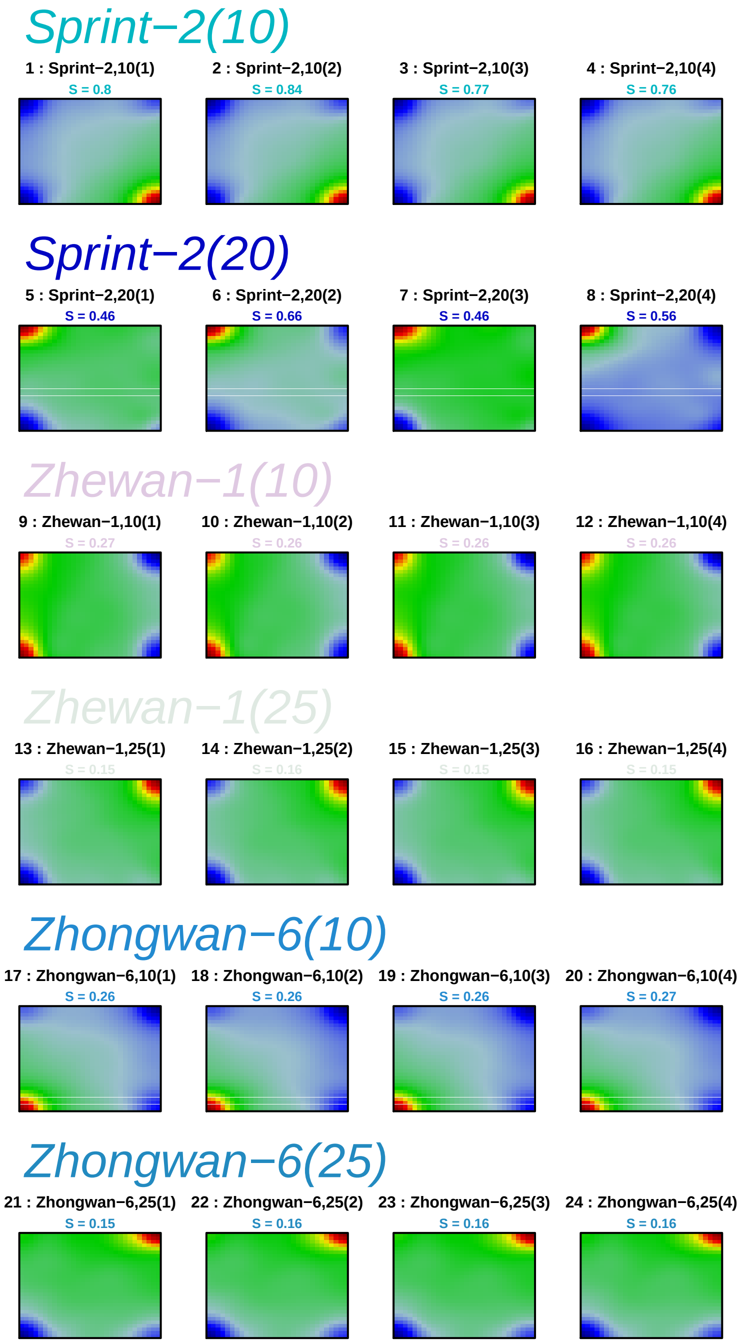

Sprint−2(10)
1 : Sprint−2,10(1)
S = 0.8
2 : Sprint−2,10(2)
S = 0.84
3 : Sprint−2,10(3)
S = 0.77
4 : Sprint−2,10(4)
S = 0.76
Sprint−2(20)
5 : Sprint−2,20(1)
S = 0.46
6 : Sprint−2,20(2)
S = 0.66
7 : Sprint−2,20(3)
S = 0.46
8 : Sprint−2,20(4)
S = 0.56
Zhewan−1(10)
9 : Zhewan−1,10(1)
S = 0.27
10 : Zhewan−1,10(2)
S = 0.26
11 : Zhewan−1,10(3)
S = 0.26
12 : Zhewan−1,10(4)
S = 0.26
Zhewan−1(25)
13 : Zhewan−1,25(1)
S = 0.15
14 : Zhewan−1,25(2)
S = 0.16
15 : Zhewan−1,25(3)
S = 0.15
16 : Zhewan−1,25(4)
S = 0.15
Zhongwan−6(10)
17 : Zhongwan−6,10(1)
S = 0.26
18 : Zhongwan−6,10(2)
S = 0.26
19 : Zhongwan−6,10(3)
S = 0.26
20 : Zhongwan−6,10(4)
S = 0.27
Zhongwan−6(25)
21 : Zhongwan−6,25(1)
S = 0.15
22 : Zhongwan−6,25(2)
S = 0.16
23 : Zhongwan−6,25(3)
S = 0.16
24 : Zhongwan−6,25(4)
S = 0.16

Sprint−2(10)

1 : Sprint−2,10(1) **2 : Sprint−2,10(2)** **3 : Sprint−2,10(3)** **4 : Sprint−2,10(4)**

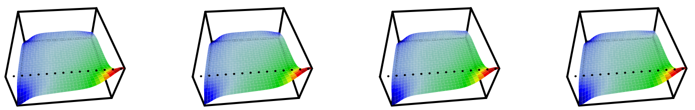

Sprint−2(20)

5 : Sprint−2,20(1) **6 : Sprint−2,20(2)** **7 : Sprint−2,20(3)** **8 : Sprint−2,20(4)**

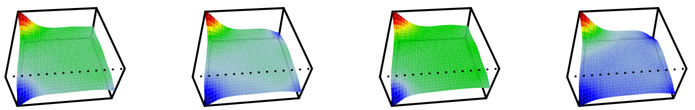

Zhewan−1(10)

9 : Zhewan−1,10(1) **10 : Zhewan−1,10(2)** **11 : Zhewan−1,10(3)** **12 : Zhewan−1,10(4)**

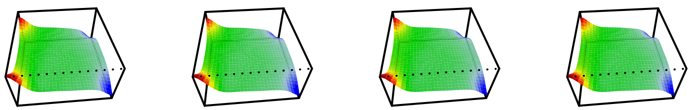

Zhewan−1(25)

13 : Zhewan−1,25(1) **14 : Zhewan−1,25(2)** **15 : Zhewan−1,25(3)** **16 : Zhewan−1,25(4)**

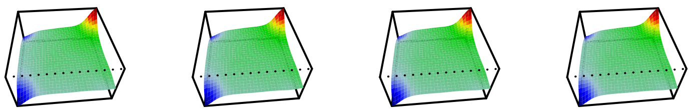

Zhongwan−6(10)

17 : Zhongwan−6,10(1) **18 : Zhongwan−6,10(2)** **19 : Zhongwan−6,10(3)** **20 : Zhongwan−6,10(4)**

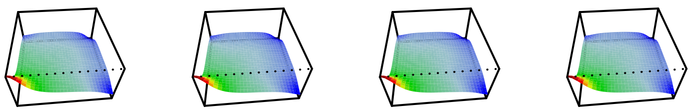

Zhongwan−6(25)

21 : Zhongwan−6,25(1) **22 : Zhongwan−6,25(2)** **23 : Zhongwan−6,25(3)** **24 : Zhongwan−6,25(4)**

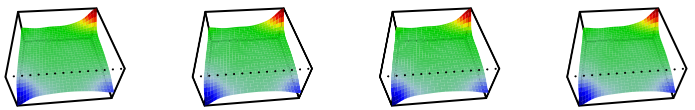

Supplement: Supplementary file 1 [file cells-09-00779-s001.zip › Supplementary materials/FileS4.zip!/FileS3/TFs - Results/Expression Portraits.pdf]
